# Quantitative Measurement and Analytical Modeling of Terahertz Wave Transmission in Natural Rock Materials Under Drying–Wetting Cycles

**DOI:** 10.3390/ma19102085

**Published:** 2026-05-15

**Authors:** Yinghu Li, Qiangling Yao, Kaixuan Liu, Minkang Han, Qiang Xu, Ze Xia

**Affiliations:** 1 State Key Laboratory for Fine Exploration and Intelligent Development of Coal Resources, China University of Mining and Technology, Xuzhou 221116, China; yinghu.li@cumt.edu.cn (Y.L.); ts23020085a31@cumt.edu.cn (K.L.); ts24020127p31@cumt.edu.cn (M.H.); xuqiangck@cumt.edu.cn (Q.X.); cumt_xiaze@cumt.edu.cn (Z.X.); 2 School of Mines, China University of Mining and Technology, Xuzhou 221116, China

**Keywords:** terahertz time-domain spectroscopy, natural rock materials, drying–wetting treatment, attenuation coefficient, non-destructive evaluation

## Abstract

**Highlights:**

- Multi-parameter characterization of THz transmission behavior in rock samples.
- Mineral composition and pore structure jointly regulate THz propagation in rocks.
- THz transmission visualizes the structure of rock samples under drying–wetting cycles.

**Abstract:**

The functional performance and structural integrity of natural rock materials under fluctuating environmental stressors are pivotal for their advanced applications. As a non-ionizing and radiation-free technology, terahertz (THz) spectroscopy offers a safe and promising alternative for non-destructive testing (NDT), uniquely capable of being deployed in open and unshielded environments. However, limited penetration depth, exacerbated by both the dense geological matrix and the extreme sensitivity of THz waves to moisture states, has long hindered its widespread application in rock characterization. This study establishes a quantitative Terahertz Time-Domain Spectroscopy (THz-TDS) framework to characterize four lithologies under drying–wetting cycles. Exponential signal attenuation across thicknesses was quantified based on the Beer–Lambert law, with attenuation coefficients ranging from 0.15 to 0.74 per millimeter. Planar transmission imaging successfully visualizes lithologic and moisture-dependent heterogeneity: limestone exhibits a dense, homogeneous structure with stable amplitude distribution; sandstone and purple sandstone show parallel statistical trends, reflecting uniform pore networks; and granite demonstrates the most pronounced imaging contrast under varying moisture states, driven by complex grain-boundary scattering. The findings reveal that THz transmission is dictated by the synergistic effects of mineral compositions and pore structures: scattering at grain boundaries and fractures leads to significant energy dissipation, whereas clay-rich lithologies exhibit the highest sensitivity to moisture variations due to water adsorption and interfacial polarization effects. As an exploration of THz technology in the non-destructive evaluation of rock materials, these findings establish an analytical framework for the quantitative assessment of microstructure evolution.

## 1. Introduction

As a key load-bearing and decorative material in civil infrastructure and architectural heritage, natural rocks exhibit mechanical behavior—including load-bearing capacity, deformation modulus, and long-term durability—that is jointly controlled by their natural structural planes, pore–fracture system, moisture state, and mineral composition [1,2]. Among these, the moisture state directly governs the weathering and degradation of construction rocks, potentially inducing structural instability and aesthetic deterioration of construction facades [3,4,5]. Therefore, establishing a detection system capable of accurately characterizing, in real time and in situ, the structural and moisture states of rock materials is of fundamental and practical importance for quantitative construction material selection, structural service-life assessment, and the long-term conservation of rock-based construction envelopes.

Traditional testing methods mainly rely on drilling and destructive laboratory experiments, which are limited in achieving in situ and continuous monitoring. In recent years, non-destructive, repeatable, and real-time testing techniques have attracted increasing attention, including computed tomography (CT), ultrasound, electromagnetic waves, electrical resistivity tomography (ERT), and nuclear magnetic resonance (NMR) [6,7,8]. Despite their widespread use, these techniques encounter inherent bottlenecks when characterizing moisture in complex porous media: ultrasonic methods are affected by anisotropy and pore–fluid coupling and require complex poroelastic models for inversion [9]; resistivity and electromagnetic induction methods exhibit limited quantitative accuracy in strongly heterogeneous rocks [10]; infrared and visible optical methods are easily influenced by water absorption, scattering, and environmental humidity [11,12]. To overcome these limitations, research has increasingly focused on multimodal fusion, including ultrasound, NMR, CT and electromagnetics, and machine learning-based multi-source inversion approaches [13,14]. Nevertheless, for engineering applications, the development of a robust, non-contact, high-sensitivity, and quantitative moisture identification system remains a pressing need.

The THz frequency band (0.1–10 THz) occupies a unique spectral niche between microwave and infrared radiation, offering both electromagnetic penetration and high sensitivity to polar molecules. Owing to its unique absorption response to water molecules, THz radiation can detect subtle moisture variations and distinguish different moisture states [8,15]. As an emerging non-contact, non-destructive testing technique, THz technology requires no coupling agents, offers high temporal and spatial resolution, and can reveal both structural heterogeneity and depth information, making it well suited for multi-scale building material characterization [16,17,18]. Existing studies have demonstrated that THz spectroscopy can accurately identify the composition and structural features of coal, rock, and soil. In coal–rock identification, significant differences in absorption coefficient, refractive index, and dielectric properties among lithotypes enable recognition accuracies up to 100% using PCA–SVM algorithms [19]; when combined with neural networks, THz spectra allow real-time inversion of coal–rock mixing ratios and shearer cutting depths, supporting intelligent mining operations [20]; and the complex refractive index of coal shows a linear relationship with moisture, enabling moisture correction and quality monitoring [21].

Beyond laboratory standards, THz technology has been successfully applied to characterize the complex mineral structures of various geological materials. The absorption and refraction features of mineral-bearing substrates within 0.2–1.2 THz show strong correlations with internal composition, enabling the non-destructive evaluation of material heterogeneity [22,23]. High-resolution THz-TDS imaging has also been utilized to map dielectric anisotropy and the distribution of internal components, achieving results consistent with X-ray CT while maintaining errors below 6% [24,25]. In the context of infrastructure and civil engineering, THz spectroscopy reveals coupled absorption–refraction behaviors among diverse natural building rocks (granite, limestone, sandstone, etc.), reflecting essential variations in mineralogy and porosity [26]. Measurements of THz optical constants in montmorillonite and carbonate rocks have further verified its capability to map dielectric response and porosity at the molecular scale [27,28]. In geotechnical applications, SVM-based THz spectral models have been used to predict the weathering depth of red-bed mudstone with errors below 7.1% [29], while THz extinction coefficients have been shown to reflect soil grain-size and moisture variation, consistent with the Lambert–Beer law and effective medium models [30].

However, existing studies have not yet quantitatively elucidated the THz response mechanisms and inter-lithologic differences in natural rocks used in construction, particularly under the coupled influence of environmental moisture states and complex pore structures. The challenge of signal attenuation in dense media also necessitates the development of more precise analytical models. To address this gap, this study investigates four representative lithologies for building applications—limestone, sandstone, purple sandstone, and granite—under five drying–wetting conditions and four thickness levels. By employing both single-point transmission and planar scanning tests, key time-domain parameters (peak amplitude, propagation delay, and waveform broadening) were quantitatively extracted. Specifically, an exponential decay model based on the Beer–Lambert law—incorporating a dynamic attenuation coefficient—was formulated to quantify THz energy loss and moisture–structure interactions in a unified manner. Furthermore, the spatial and statistical features of planar amplitude imaging were analyzed in conjunction with mineralogical and microstructural data to elucidate the dominant factors controlling THz propagation and energy dissipation in porous rocks. These findings provide robust theoretical and experimental support for advancing quantitative NDT and structural health monitoring of rock materials.

## 2. Experimental Materials and Methods

### 2.1. Sample Preparation and Moisture Conditioning

The rock specimens used in this study were collected from representative lithologies in different regions, including limestone, purple sandstone, sandstone, and granite. To ensure geometric precision and testing reliability, the specimens were first finely processed using a precision grinding machine so that the upper and lower surfaces were smooth and parallel. The thickness of each specimen was then measured with a digital vernier caliper, and the measurement error was controlled within ±0.05 mm.

**Figure 1:**
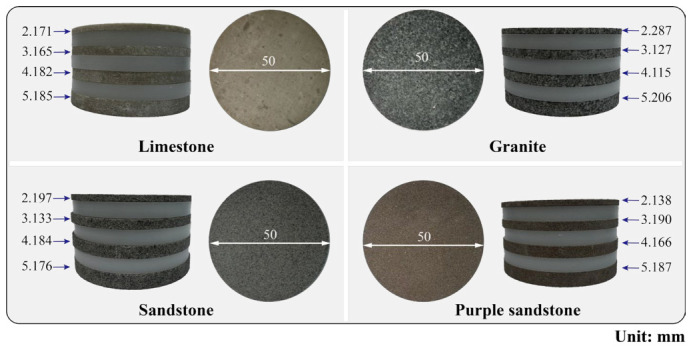
Specimens of four lithologies with different thicknesses.

Cylindrical rock slices with a diameter of 50 mm and target thicknesses of 2 mm, 3 mm, 4 mm, and 5 mm were prepared. Considering the potential dimensional deviations during actual processing, the nominal and measured thicknesses of each specimen were cross-checked and recorded; the actual thicknesses are presented in Table 1. To further improve measurement accuracy, an electronic analytical balance (accuracy 0.1 mg) and a digital micrometer (accuracy 0.001 mm) were employed during the machining and inspection processes. After processing, all specimens were sealed with plastic film to prevent moisture variation. The prepared rock samples are shown in Figure 1.

To investigate the effect of drying–wetting treatment on the terahertz transmission characteristics of rock specimens, specimens of different thicknesses were subjected to a series of conditioning stages, including: natural state (untreated), initial drying for 6 h, initial soaking for 6 h, secondary soaking for 6 h, and secondary drying for 12 h. All drying processes were conducted in a thermostatic oven at 50 °C, ensuring controlled temperature to prevent cracking of thin specimens caused by rapid moisture loss. The soaking processes were performed in a room-temperature saturation chamber to maintain consistent environmental conditions. After each treatment, terahertz time-domain transmission spectroscopy tests were carried out immediately.

As shown in Table 1, the mass and density evolution of specimens with different lithologies during the drying–wetting treatments exhibited differences. The limestone specimens had the highest density, ranging from 2645 to 2670 kg/m^3^, with a total variation of only 0.30% after two cycles of soaking and drying, indicating an extremely compact internal structure with very low porosity and limited water absorption and desorption capacity. The granite specimens also exhibited relatively high densities (2826–2850 kg/m^3^) with minimal overall variation, reflecting their low-porosity characteristics. In contrast, sandstone and purple sandstone showed lower natural densities, approximately 2700 kg/m^3^ and 2400 kg/m^3^, respectively. Their density variations before and after the drying–wetting treatments were about 1.91% and 6.44%, respectively, suggesting stronger reversible water absorption–desorption behavior and higher pore connectivity. Moreover, the density differences among specimens of the same lithology under identical treatment conditions but with different thicknesses were relatively small, indicating good overall homogeneity of the specimens and a limited influence of thickness on the overall pore distribution.

### 2.2. THz-TDS Testing Procedure

**Figure 2:**
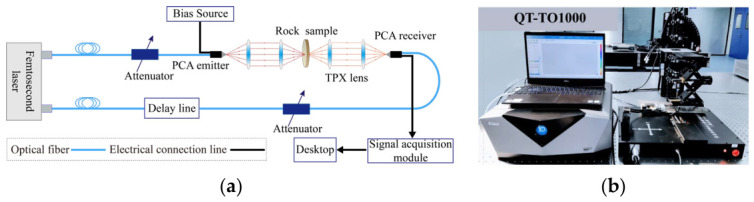
Terahertz time-domain transmission spectroscopy (THz-TDS) system: (**a**) schematic diagram; (**b**) physical photograph.

In this experiment, terahertz tests on rock specimens were conducted using a QT-TO1000 terahertz time-domain spectroscopy/3D imaging system manufactured by Qingyuan Fengda Terahertz Technology Co., Ltd. (Qingdao, China). The system employs a femtosecond laser with a central wavelength of 1550 nm as the light source, delivering a pulse width of less than 100 fs and a repetition rate of 80 MHz. The laser beam is transmitted through an optical fiber to the terahertz transmitting antenna, where terahertz pulse signals are generated under a bias voltage. The emitted pulse is collimated and focused by TPX lenses before penetrating the specimen under test. The transmitted terahertz signal is then collected by symmetrically arranged TPX lenses and delivered to the receiving antenna. The receiving antenna converts the terahertz signal into a photoelectric signal, which is subsequently processed and displayed through the signal acquisition module and the upper-level control system. The optical path layout and physical structure of the system are shown in Figure 2.

The scanning frequency range of the system is 0.1–4.5 THz, with a spectral resolution of 8 GHz and a dynamic range exceeding 75 dB. It is capable of detecting specimens with thicknesses ranging from 30 μm to 9 mm. The imaging module allows angle adjustment within 40–180°, supports both transmission and reflection modes, and is equipped with a compact two-dimensional scanning and panning stage (imaging field of view: 100 mm × 100 mm; maximum scanning speed: 60 pixels/s), enabling acquisition of high-quality two-dimensional and three-dimensional terahertz imaging data.

**Figure 3:**
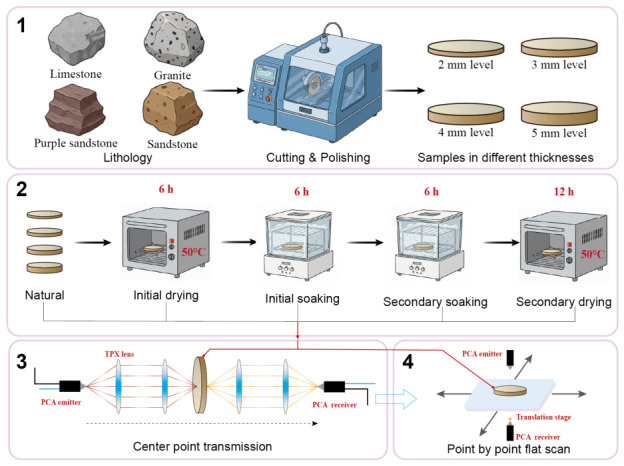
Experimental procedure: specimen preparation, saturation and drying treatment, and THz testing.

During the experiment, the femtosecond laser pulse was divided into two beams: a pump beam and a probe beam. The pump beam drove the transmitter to generate the terahertz (THz) pulse, which, after transmitting through the specimen, interacted with the probe beam at the receiving antenna to obtain the time-domain THz signal carrying information about the specimen’s internal structure. To ensure measurement accuracy, the optical path and translation stage were carefully calibrated and adjusted so that the center of the specimen was precisely aligned with the focal point of the THz beam. The experimental environment was maintained at a temperature of 24 °C and a relative humidity below 10%. During testing, both central-point transmission and planar scanning measurements were performed for each specimen under different drying–wetting conditions. In the central-point transmission mode, the specimen was fixed on a movable platform, and its height was adjusted to obtain the optimal THz transmission signal, followed by a single-point measurement at the specimen center. In the planar scanning mode, the X–Y translation stage was controlled to move point by point with a step size of 0.2 mm, enabling two-dimensional surface scanning of the specimen. The overall experimental procedure of this study is illustrated in Figure 3. Gemini 3.1 was utilized to assist in generating the schematic representations of different lithological blocks and the experimental apparatus, including the drying oven, soaking chamber, and cutting and polishing equipment.

## 3. Results and Discussion

### 3.1. Time-Domain Signal Characteristics of Rock Specimens with Different Thicknesses Under Single-Point Transmission

To investigate the influence of specimen thickness and lithology on THz wave propagation, this section systematically analyzes the time-domain THz transmission characteristics of limestone, sandstone, purple sandstone, and granite specimens under the natural state.

To clarify the physical meaning of the characteristic peaks in the time-domain waveform, the air reference waveform was introduced as the temporal reference. The main peak position of the air reference waveform was defined as *t*_air_, while the main peak position of the sample transmission waveform was defined as *t*_1_. For an approximately homogeneous slab sample, the delay of the reflection generated at the back surface of the sample relative to the main sample peak can be approximated as twice the delay of the sample main peak relative to the air reference main peak. Therefore, the theoretical position of the second characteristic peak, *t*_2_, can be expressed as:

(1) $$t_{2}=t_{1}+2\left(t_{1}-t_{\mathrm{air}}\right)$$

**Figure 4:**
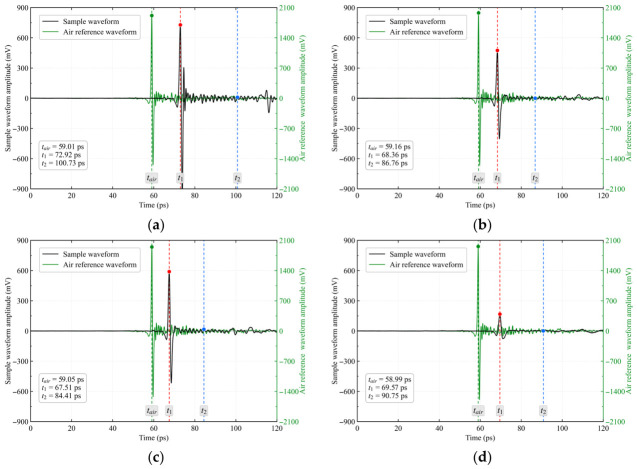
Echo identification results for 2-mm-thick rock samples of different lithologies in their natural state. (**a**) limestone, (**b**) sandstone, (**c**) purple sandstone, (**d**) granite.

As shown in Figure 4, the echo identification results of the 2-mm-thick natural rock samples exhibit lithology-dependent differences. The sample peaks of all rock specimens were delayed relative to the air reference main peak. Limestone exhibited the largest peak delay, reaching 13.91 ps, whereas the delays for granite, sandstone, and purple sandstone were 10.59, 9.20, and 8.45 ps, respectively. These results indicate that, under the same thickness condition, lithological differences alter the effective propagation time of the terahertz pulse, reflecting variations in effective optical path length, group velocity, and internal structure among the rock specimens. The *t*_2_ position derived from the two-fold delay relation further characterizes the cumulative propagation delay of different specimens. The normalized amplitudes at this position were generally low, suggesting weak waveform responses at the corresponding time and limited contributions from subsequent echoes or residual oscillations.

The echo-identification results indicate that the THz time-domain signals of different lithological samples contain a main transmitted peak followed by varying degrees of subsequent responses. To further quantify the effect of sample thickness on THz wave propagation, the peak amplitude, peak delay, and waveform width of rock samples with different thicknesses were analyzed.

**Figure 5:**
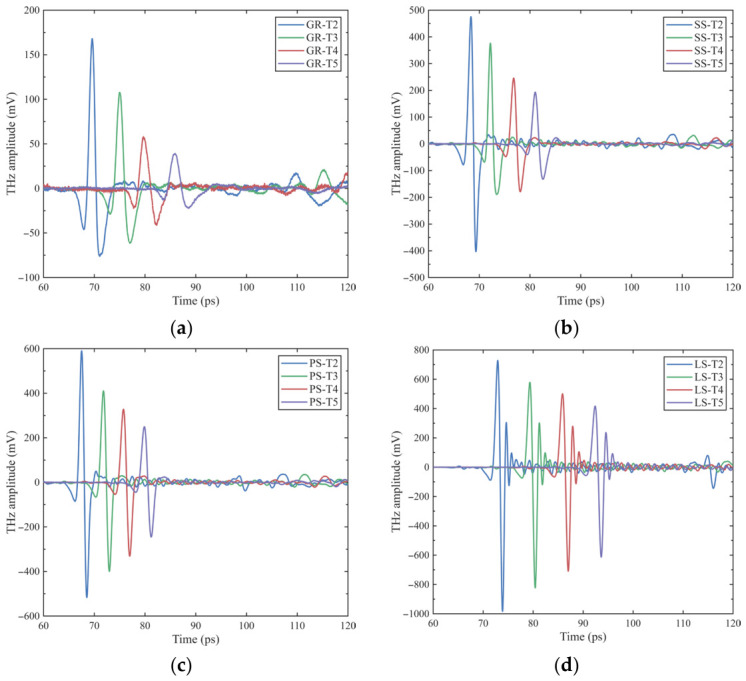
THz transmission time-domain waveforms of specimens with four lithologies at different thicknesses (Reference value: 2203.71): (**a**) limestone, (**b**) sandstone, (**c**) purple sandstone, (**d**) granite.

As shown in Figure 5, when the specimen thickness increases from 2 mm to 5 mm, all four lithologies exhibit consistent variation trends in their time-domain waveforms. The main peak progressively lags, and the amplitude decreases significantly. The waveform continuously broadens, accompanied by a more prominent secondary response following the main transmitted peak. This indicates that the increased thickness extends the effective propagation path of THz waves within the medium, subjecting them to greater influence from mineral grains, pore structures, and internal heterogeneities. Consequently, scattering, absorption, and phase delay effects are intensified, manifesting as more apparent energy attenuation and temporal broadening.

Clear lithological differences are observed under this thickness effect: limestone exhibits the highest baseline transmission amplitude at a smaller thickness and the largest increase in main peak delay with thickness, indicating a higher effective refractive index. Granite shows the lowest overall transmission amplitude, with more pronounced waveform distortion and broadening as thickness increases, implying the strongest internal absorption, scattering, and dispersion. Sandstone and purple sandstone fall between these two extremes, showing smaller time delays and moderate amplitudes, reflecting their relatively loose structures and lower effective refractive indices. To further quantify these characteristics, the attenuation of the main peak amplitude, time delay, and waveform broadening is analyzed separately.

**Figure 6:**
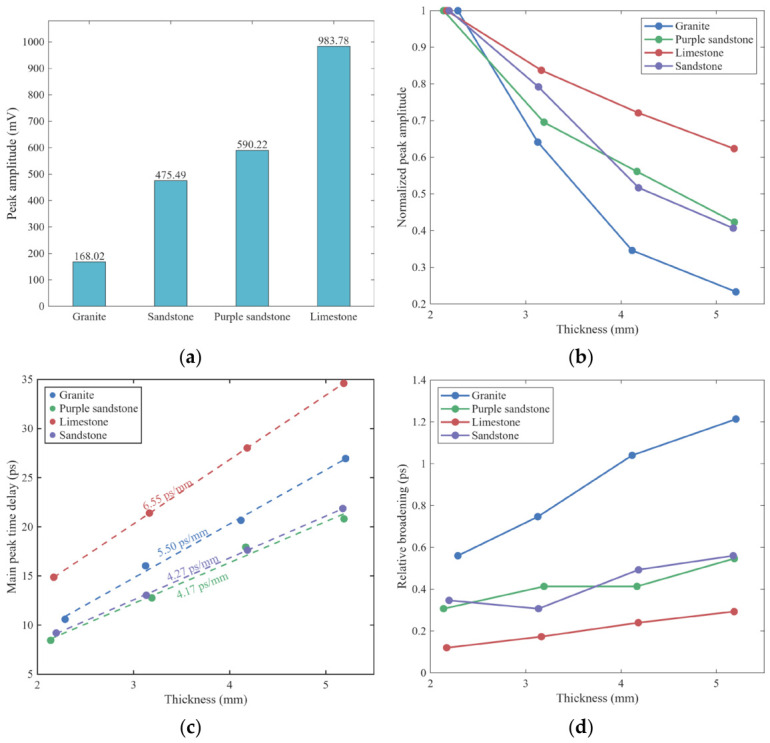
Variation characteristics of THz time-domain signals of specimens with four lithologies under different thicknesses: (**a**) Peak amplitude at the 2-mm-thick group; (**b**) normalized attenuation of peak amplitude with increasing thickness (referenced to the 2-mm-thick group); (**c**) linear fitting of main peak time delay versus thickness; (**d**) variation in relative waveform broadening with thickness.

As shown in Figure 6a, the peak amplitude of limestone is the highest (983.78 mV), which is 5.85 times that of granite (168.08 mV) and significantly higher than that of purple sandstone (590.22 mV) and sandstone (475.49 mV). This initial difference reflects the combined influence of mineral composition, pore structure, and moisture state on THz wave absorption and scattering. With increasing specimen thickness, the main peak amplitude exhibits continuous attenuation, as illustrated in Figure 6b. The amplitude of all specimens decreases monotonically, though the decay rates vary significantly. Granite shows the most rapid energy loss, retaining only 18.21% of its initial amplitude at the 5-mm-thick group, while limestone displays the slowest attenuation, with a retention ratio as high as 58.65%. Sandstone and purple sandstone fall between these two, both maintaining amplitudes above 42%.

The main peak time delay increases approximately linearly with thickness (see Figure 6c), consistent with the linear propagation-delay relationship in a homogeneous medium. Linear fitting results show that limestone has the largest delay coefficient per unit thickness (6.59 ps/mm), indicating the strongest deceleration effect on THz waves and the highest effective refractive index. Granite ranks second (5.37 ps/mm), while purple sandstone (4.26 ps/mm) and sandstone (4.23 ps/mm) exhibit the smallest and nearly identical values, suggesting relatively loose internal structures with a weaker influence on propagation velocity. These results indicate that the refractive behavior of specimens is primarily governed by their compactness and pore characteristics.

The relative waveform broadening characteristics of different lithologies also exhibit significant differences (Figure 6d). It should be noted that, since the reference signals and all sample signals in this study were collected under identical optical system and focusing conditions, the inherent optical aberrations of the optical system exist as a constant systematic bias in each set of tests; therefore, the focus here is on analyzing the relationship between sample variations and the relative changes in the amount of broadening. With increasing thickness, waveform broadening continuously increases for all rocks, but the growth rate varies. Granite exhibits the largest broadening, from 0.56 ps at 2 mm to 1.21 ps at 5 mm, implying that its multiphase interfaces and microfractures cause the strongest dispersion and multiple-scattering effects. In contrast, limestone shows the smallest broadening (only 0.29 ps at the 5-mm-thick group), indicating a uniform and compact structure with minimal waveform distortion. Sandstone and purple sandstone exhibit intermediate and similar broadening magnitudes, suggesting comparable pore distribution and modulation effects.

These results indicate that lithology-dependent THz amplitude, delay, and waveform characteristics are jointly governed by rock compactness, pore structure, and mineral composition. Limestone exhibits the highest amplitude and the greatest delay, indicating the densest structure and lowest porosity, with minimal energy loss but the slowest propagation velocity—representing a typical high-transmission, high-refraction characteristic. Although granite has a high density, its multiphase crystal interfaces and microfractures result in strong absorption and multiple scattering, leading to significant energy attenuation and waveform distortion. Sandstone and purple sandstone exhibit intermediate amplitudes and delays; their well-developed and connected pores cause greater energy loss but faster propagation. Notably, purple sandstone, enriched in clay minerals, exhibits slightly lower amplitude than sandstone due to its stronger water-adsorption capacity.

### 3.2. Relationship Between Main-Peak Amplitude and Thickness of Specimens Under Different Moisture States and Model Fitting

To reveal the frequency-domain response characteristics of rock THz-TDS signals under dry and wet conditions, this section analyzes variations in spectral energy distribution for samples with different lithologies, thicknesses, and moisture states. Particular attention is given to shifts in the dominant PSD peak frequency and differences in the normalized power spectra, with the aim of clarifying the effects of thickness, moisture, and lithological heterogeneity on high-frequency attenuation and the low-frequency shift in THz signals.

To analyze the frequency-domain response characteristics of rock samples under different thicknesses and wetting–drying conditions, a Fast Fourier Transform (FFT) was performed on the acquired THz time-domain signal E(t). This process transforms the signal from the time domain to the frequency domain, yielding the corresponding complex spectrum E(f). Theoretically, the continuous complex spectrum E(f) is expressed as:

(2) $$E(f)=\int_{-\infty }^{+\infty }E(t)e^{-i2\pi ft}dt$$

Here, f is the frequency and t is the time.

To quantitatively evaluate the frequency-domain response of rock samples under various thicknesses and moisture states, the dominant peak frequency of the Power Spectral Density (PSD peak frequency) was introduced as a characteristic parameter to represent changes in the spectral energy distribution of the THz-TDS signals. A Hann window function was applied to the acquired THz time-domain signals, followed by the FFT to obtain the frequency-domain signal E(f). Subsequently, the Power Spectral Density (PSD) was calculated as follows:

(3) $$PSD(f)=\frac{|E(f){|}^{2}}{f_{s}\sum_{n=1}^{N}w_{n}^{2}}$$

Here, $f_{s}$ is the sampling frequency, $w_{n}$ represents the discrete sequence of the Hann window function, and N is the total number of sampling points.

**Figure 7:**
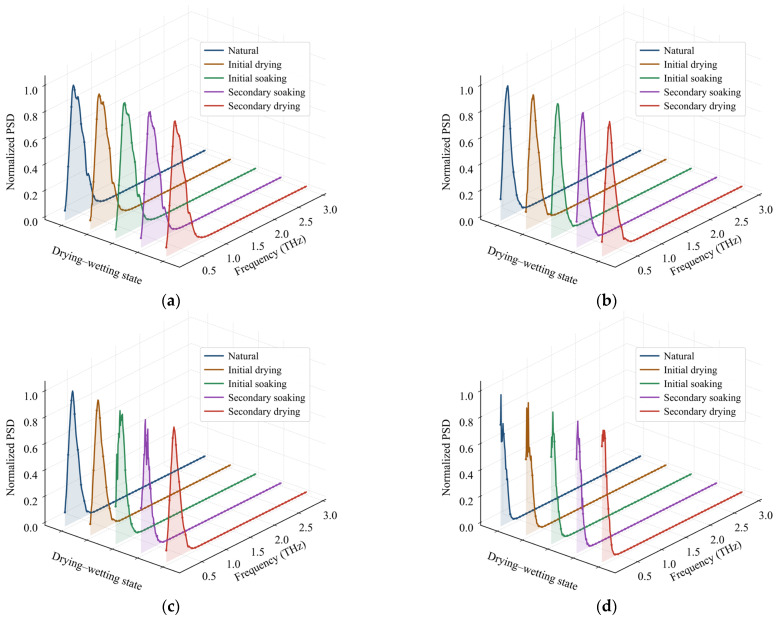
Frequency-domain PSD power spectral distribution for different lithologies and moisture states: (**a**) limestone, (**b**) sandstone, (**c**) purple sandstone, (**d**) granite.

As shown in Figure 7, the THz time-domain signals of rock specimens in the 5-mm-thick group under different lithologies and moisture conditions were transformed by FFT to obtain the normalized frequency-domain power spectra. The differences in PSD distributions among lithologies may be associated with variations in mineral composition, grain structure, and internal pore characteristics. Sandstone exhibits relatively broad spectral peaks, indicating a more dispersed distribution of frequency-domain energy. Purple sandstone shows more pronounced fluctuations in peak morphology, suggesting a higher degree of spectral complexity. Limestone presents a more concentrated main peak and relatively smooth spectral profiles, whereas granite is characterized by sharper and more prominent peaks, with frequency-domain energy more strongly concentrated around the dominant peak. In contrast, for specimens of the same lithology, the PSD curves under different drying–wetting states are generally similar and do not exhibit distinct, isolated, and stable narrow-band absorption features. This indicates that the influence of moisture-state variation on the spectra is not manifested as independent absorption peaks at specific frequencies, but rather as an overall redistribution of spectral energy, shifts in the dominant peak frequency, and attenuation of high-frequency components.

**Figure 8:**
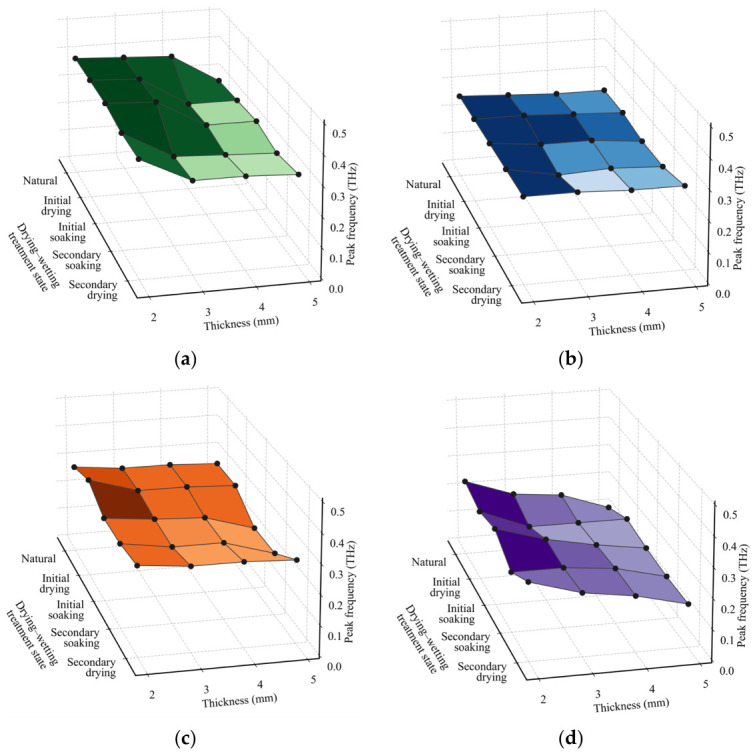
PSD peak frequency under different lithologies, thicknesses, and moisture states: (**a**) limestone, (**b**) sandstone, (**c**) purple sandstone, (**d**) granite.

As shown in Figure 8, the dominant PSD peak frequencies were analyzed for rock specimens with different lithologies, thicknesses, and moisture conditions. With increasing specimen thickness, the dominant PSD peak frequency generally shifts toward lower frequencies, indicating that a greater propagation path enhances the attenuation of high-frequency THz components within the rock. Variations in moisture conditions further affect the spectral energy distribution. After saturation treatment, the dominant PSD peak frequency generally decreases, with the most pronounced low-frequency shift observed under the 12 h saturated condition. After secondary drying, the dominant peak frequency partially recovers; however, residual differences remain, suggesting that drying–wetting cycling has an incompletely reversible effect on the frequency-domain response of the rocks. Distinct differences are also observed among lithologies in the evolution of the dominant PSD peak frequency. Limestone exhibits relatively higher dominant peak frequencies overall and is more sensitive to thickness variation. Granite shows the lowest dominant peak frequencies and the most evident low-frequency shift. Sandstone presents a comparatively gradual variation, whereas purple sandstone exhibits a pronounced moisture-sensitive response under saturated conditions.

To further quantitatively describe the attenuation law of the time-domain peak amplitude for samples under different moisture states, the classic Beer–Lambert model [31,32] was employed to fit the experimental data. The model can be expressed as follows:

(4) $$P_{a}=P_{0}\exp\left(-\alpha T\right)$$

Here, *α* is the effective attenuation coefficient (mm^−1^), *P_a_* is the peak amplitude measured at a sample thickness of *T* (mV), *T* is the specimen thickness (mm), and *P*_0_ is the reference peak amplitude measured in air (mV). This model is used to predict the actual testing thickness of the specimen:

(5) $$T=-\frac{1}{\alpha }\ln\frac{P_{a}}{P_{0}}$$

**Figure 9:**
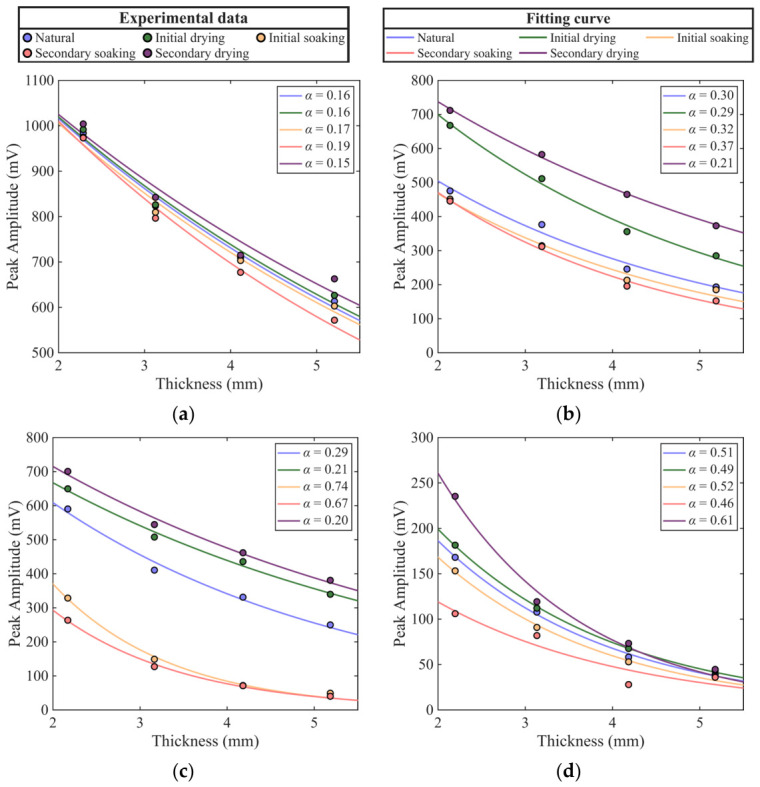
Attenuation characteristics and model fitting of the peak amplitude of rock specimens with thickness under different dry–wet treatments (**a**) limestone, (**b**) sandstone, (**c**) purple sandstone, (**d**) granite.

**Figure 10:**
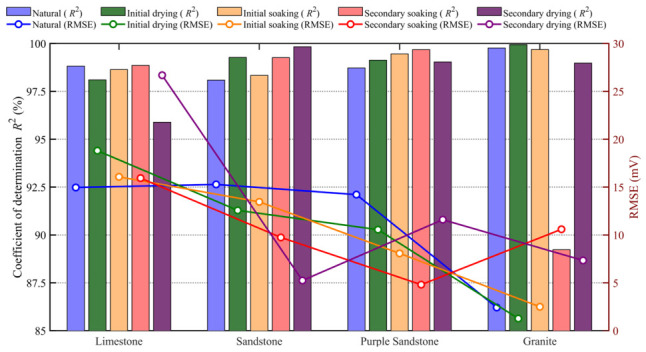
The coefficient of determination and RMSE of the fitted model for peak amplitude–thickness evolution.

The experimental results and corresponding fitting curves are shown in Figure 9, and the coefficients of determination *R*^2^ are presented in Figure 10. It can be observed that *R*^2^ for all lithologies and moisture states exceed 96%, indicating that the exponential model accurately characterizes the attenuation behavior of THz peak amplitude under different moisture states, demonstrating excellent applicability and stability.

As shown in Figure 9, the amplitude variations under different moisture states exhibit distinct patterns reflecting both moisture suppression and thickness effects. At any given thickness, the amplitude in the dried state is significantly higher than that in the soaked state, indicating the strong absorption of THz waves by water molecules. For porous lithologies such as sandstone and purple sandstone, the moisture-suppression effect is far more pronounced than the thickness effect. For example, in purple sandstone, the peak amplitude after the secondary drying at the 5-mm-thick group reaches 380.65 mV, which exceeds the values at the 2-mm-thick group under the initial (328.67 mV) and secondary (263.49 mV) saturation stages. A similar trend is observed in sandstone: the 5-mm-thick group specimen after the secondary drying shows a peak amplitude of 372.89 mV, which is considerably higher than that of the 3-mm-thick group specimen in the saturated state. In contrast, granite and limestone are less affected by moisture; in particular, limestone exhibits the smallest inter-curve spacing and the smoothest variation, reflecting its dense structure and lowest sensitivity to moisture changes.

Furthermore, for all lithologies, the peak amplitude decays rapidly with thickness in the thinner range but gradually stabilizes as thickness increases. This indicates that for thin specimens, the absorption effect of water on the transmitted signal is more pronounced, whereas for thicker specimens, intrinsic absorption and scattering within the rock become the dominant factors. Based on the fitted curves, the thickness most sensitive to moisture variation for each lithology can be identified at the point showing the largest amplitude difference between the secondary drying and secondary saturation states: granite, 0.75 mm (a difference of 291.67 mV); sandstone, 1.33 mm (a difference of 421.35 mV); purple sandstone, 1.19 mm (a difference of 703.61 mV); and limestone, 1.76 mm (a difference of 59.80 mV).

The attenuation coefficient *α* quantifies the rate of THz energy loss per unit thickness of the medium, and its evolution clearly reveals the dominant role of moisture in THz attenuation. From the natural state to the initial drying stage, *α* decreases significantly, indicating that the removal of free and partially adsorbed water from pores and microcracks substantially weakens THz absorption. From the initial drying to the initial soaking, *α* increases rapidly, reflecting the reintroduction of a strongly absorbing medium as the specimen reabsorbs water. From the initial to the secondary soaking, *α* shows only a slight increase, suggesting that the moisture content has approached or reached saturation. From the secondary soaking to the secondary drying, *α* drops sharply to its minimum, representing the lowest level throughout the entire process. The absolute range of *α* among different lithologies directly reflects their varying sensitivities to moisture changes.

The absolute range of *α* across lithologies distinctly illustrates their differential responses to moisture variation. Granite exhibits higher α values (0.49–0.61/mm) under most wetting conditions, but its variation becomes more pronounced as conditions change. Conversely, limestone has the lowest α values (0.25–0.31/mm) and the narrowest variation range. This reflects its dense and uniform structure, ensuring stable THz attenuation and minimal sensitivity to water-bearing conditions. Sandstone and purple sandstone exhibit intermediate *α* ranges, showing typical porous-medium behavior: although their attenuation magnitudes are smaller, they are highly sensitive to moisture changes, with overall THz attenuation predominantly governed by adsorbed water and interconnected pores.

**Figure 11:**
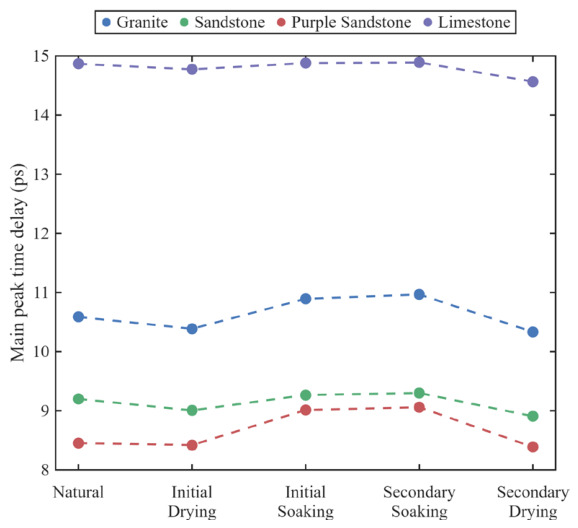
Main peak time delay characteristics of rock specimens in the 2-mm-thick group under different wetting–drying treatments.

To evaluate the damage induced during the wetting–drying cyclic treatment, the evolution patterns of the main peak time delay (TOF) for the four rock types within the 2-mm-thick group under various water treatment cycles were further analyzed, as illustrated in Figure 11. The results indicate that the TOF values consistently maintain a descending order of “limestone, granite, sandstone, and purple sandstone” throughout the entire process, highlighting the decisive influence of the intrinsic properties of the rock matrix on terahertz propagation.

In the dynamic alternation of wetting–drying cycles, the TOF exhibits high sensitivity to moisture migration and microstructural variations. First, in the initial drying stage, as the high-refractive-index free water in the pores is replaced by low-refractive-index air, coupled with the initial opening of native microcracks inside the rock caused by thermal treatment, the TOFs of all four rocks show a measurable decrease; for example, granite decreases by up to 0.2 ps. Subsequently, in the initial soaking stage, water refills the pores, and the water-induced swelling of some rock matrices leads to the closure of partial microcracks, substantially increasing the effective refractive index and prompting a significant rebound in the TOF of each specimen. Notably, the secondary soaking stage generally exhibits a longer time delay, such as the TOF of purple sandstone increasing from 8.45 ps to 9.06 ps, indicating that after the initial cycle, more damage crack seepage channels have formed inside the rock, enhancing its water retention capacity. Finally, in the secondary drying stage, the TOFs of all specimens drop to the lowest point of the entire cycle, significantly lower than the initial drying state. This phenomenon confirms that the wetting–drying cycle induces irreversible physical damage within the rock. The severe alternation of drying shrinkage and wetting expansion not only expels pore water but also catalyzes the initiation and propagation of numerous secondary microcracks in the rock matrix. These newly generated microcracks significantly increase the air volume fraction inside the rock, causing the macroscopic effective refractive index of the medium to be further reduced, which directly manifests as the shortening of the TOF.

### 3.3. Planar Scanning Imaging Based on Peak Amplitude and Its Statistical Evolution

Before performing THz transmission imaging analysis, the validity of the plane-wave approximation in the THz transmission analysis was examined. Specifically, the propagation characteristics of the THz beam at the sample position were estimated based on the experimental optical-path parameters, in order to determine whether the plane-wave approximation was applicable under the present experimental conditions. In this study, the TPX lens used in the experiment had a focal length of 50 mm and an aperture of 38.1 mm. The THz beam radius at the sample measurement position was estimated to be ω0 = 0.8 mm.

**Figure 12:**
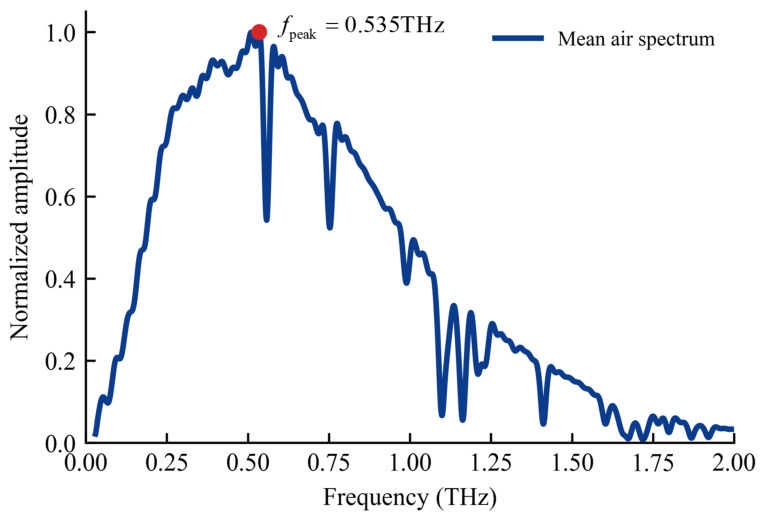
Frequency spectrum of the air reference signal.

To determine a representative frequency for the focal-length estimation, the time-domain reference signal measured in air without a sample was Fourier transformed to obtain its frequency-domain spectrum *E*_ref_(*f*). The frequency corresponding to the maximum spectral amplitude, *f*_peak_, was then selected as the representative frequency for focal-length estimation. The frequency-domain spectrum of the air reference signal is shown in Figure 12.

The confocal parameter was calculated according to the Gaussian beam relation:

(6) $$b=2z_{R}=\frac{2\pi \omega_{0}^{2}}{\lambda }$$

Here, λ is the corresponding wavelength in mm (derived from $f_{\mathrm{peak}}$). Substituting $\omega_{0}=0.8$ mm gives a confocal parameter of b=7.18 mm, which is larger than the maximum sample thickness considered in this study, 5 mm. Therefore, the sample thickness is smaller than the confocal parameter, indicating that the plane-wave approximation is generally acceptable under the present experimental conditions.

**Figure 13:**
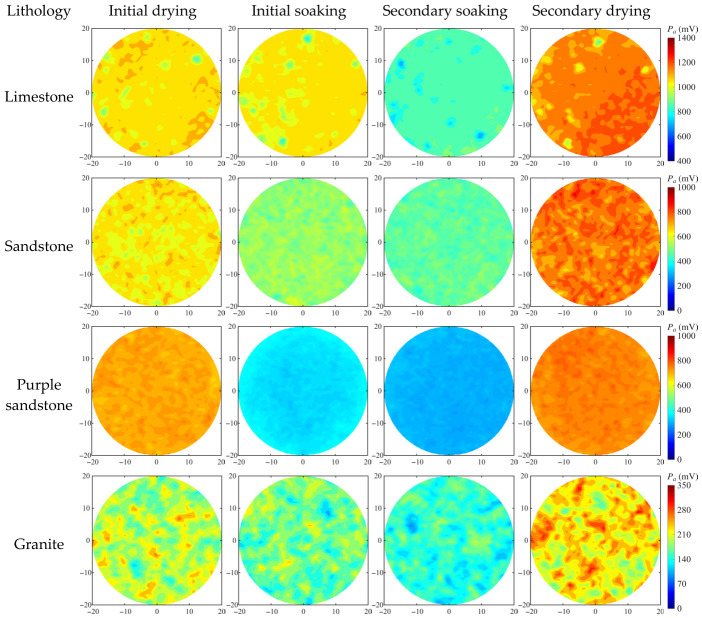
Planar imaging of time-domain main-peak amplitude for specimens of different lithologies in the 2-mm-thick group.

**Figure 14:**
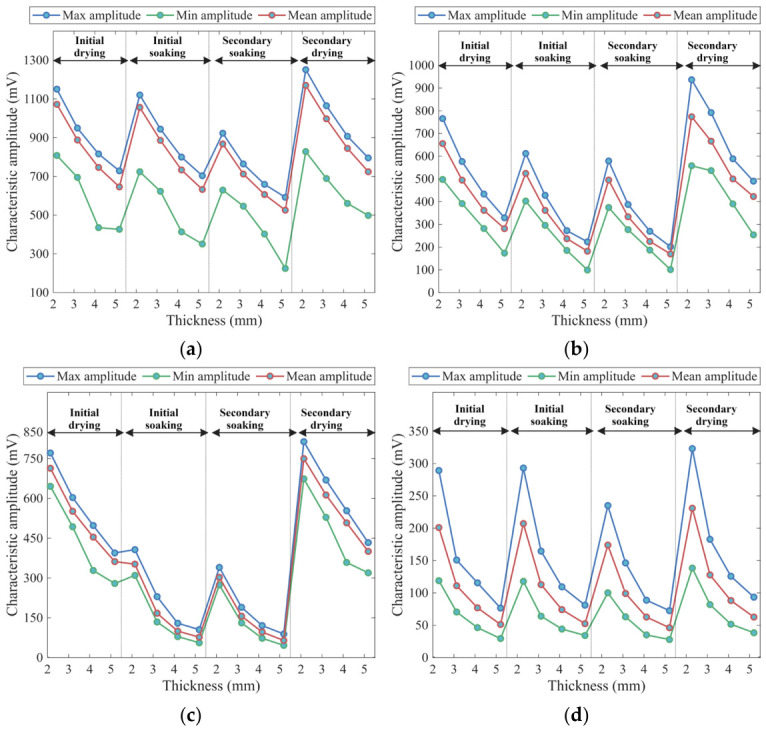
Evolution of statistical characteristics of time-domain main-peak amplitude of rock specimens with thickness and moisture treatment under planar scanning: (**a**) limestone, (**b**) sandstone, (**c**) purple sandstone, (**d**) granite.

According to the planar scanning results of the specimens, the time-domain peak amplitude at each point on the scanning plane was used for imaging analysis. To minimize the influence of edge effects and air coupling, only the region within a radius of 20 mm from the specimen center was considered for statistical analysis. Figure 13 presents the amplitude imaging results for specimens in the 2-mm-thick group, while Figure 14 illustrates the evolution of the maximum, minimum, and average peak amplitudes within the imaging plane under different thicknesses and drying–wetting conditions.

As shown in the imaging results in Figure 13, the amplitude distribution of limestone specimens is the most uniform across all stages, with only a few localized low-amplitude regions. These regions are highly sensitive to moisture variation and correspond to limited pores or microfractures, whereas the rest of the specimen exhibits high and stable amplitudes, reflecting a dense matrix structure and stable THz propagation. The impact of moisture is primarily sequestered within localized porous regions.

Sandstone specimens show pronounced spatial nonuniformity, with alternating high- and low-amplitude regions that maintain a similar pattern during both drying and soaking processes. This indicates a well-developed and highly connected pore system, allowing efficient water migration and evaporation. The amplitude distribution of purple sandstone is similar to that of sandstone but with weaker contrast between high- and low-amplitude regions. Its overall amplitude is slightly higher than that of sandstone in the natural state; however, it decays more rapidly during soaking and does not fully recover after secondary drying. This implies that the matrix is enriched with highly adsorptive clay minerals, imparting a greater water-retention capacity and retarding the evaporation process.

Granite specimens exhibit the lowest overall amplitude and a highly nonuniform spatial distribution, with multiple prominent low-amplitude zones. As saturation progresses, these zones expand and decay rapidly, indicating that internal pores and fractures act as strong absorption–scattering centers after water infiltration. Consequently, granite specimens show the most pronounced THz response variation among the tested lithologies under different moisture states.

As shown in Figure 14, the planar transmission results demonstrate that the thickness effect still follows an exponential attenuation trend, consistent with the single-point transmission results. The influence intensity of drying–wetting treatment is clearly controlled by lithology. Specifically, limestone exhibits the smallest spacing between its maximum and average amplitude curves, always below 90 mV and nearly parallel, indicating a uniform and dense structure with most regions maintaining high amplitude levels. The variation pattern of the minimum values is less distinct, mainly affected by limited pores, microfractures, and fine-scale heterogeneity. In the 2-mm-thick group, the maximum amplitude decreases by 30.29 mV and 197.33 mV after the initial and secondary soaking stages, respectively, suggesting that the water infiltration process in the limestone matrix is slow and the connectivity of pores and fractures is limited.

For sandstone and purple sandstone, the spacing between average and extreme amplitude curves remains generally consistent, indicating relatively uniform pore distributions. The amplitude variations induced by drying–wetting cycles are more pronounced—particularly in purple sandstone, where the curve fluctuations during the natural–saturated–dried alternations are the most distinct, with a maximum amplitude difference of 474.4 mV, reflecting its strong moisture sensitivity.

Granite also shows a relatively uniform pore distribution, but with larger pore sizes, resulting in greater spacing between the average and extreme amplitude curves compared to other lithologies. The difference between its maximum and minimum values becomes more pronounced with increasing thickness, indicating that granite exhibits ‘thickness-dominant’ attenuation characteristics, in contrast to the ‘moisture-sensitive’ behavior observed in more porous lithologies.

### 3.4. Influence of Composition, Morphology, and Structure on THz Transmission Characteristics

To further clarify the mechanisms by which lithology and structure affect THz response, additional mineralogical and microstructural analyses were conducted based on the THz time-domain test results. X-ray diffraction (XRD) was used to determine mineral composition, while small fragments from the same specimen batch were examined using scanning electron microscopy (SEM) for surface morphology and computed tomography (CT) for internal structural imaging.

**Figure 15:**
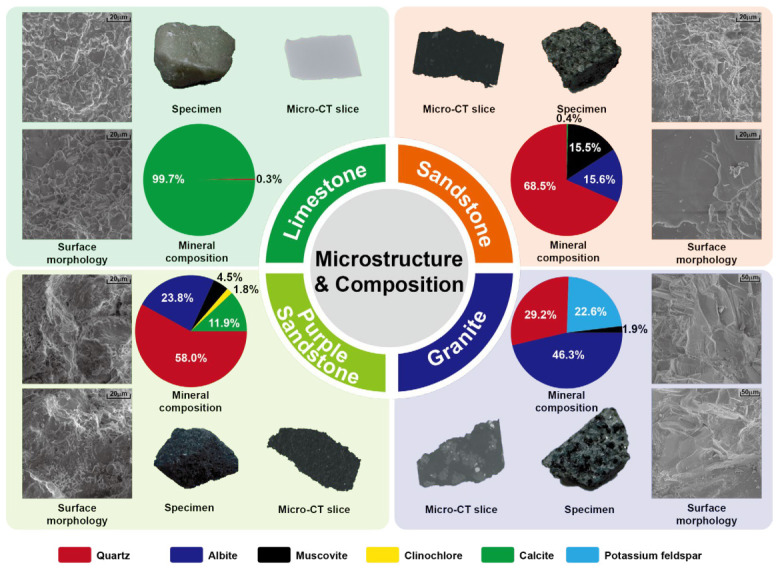
Integrated characterization of rock microstructure and mineral composition.

As shown in Figure 15, the integrated characterization results for the four lithologies reveal distinct mineralogical and structural features. Limestone is primarily composed of calcite as a monomineralic mineral. SEM micrographs reveal a dense, interlocking mosaic-like microstructure, and CT slices display uniform grayscale, indicating a low density of scattering sources. Consequently, the limestone specimens exhibit superior transmission performance, weaker sensitivity to drying–wetting treatment, and more pronounced time-delay growth with increasing thickness, showing an overall stable response.

Sandstone consists mainly of a quartz framework with feldspar and a certain proportion of muscovite. SEM and CT images reveal connected pore throats and fine interlaminar fissures, resulting in moderate scattering and multipath effects. Therefore, its attenuation and broadening levels are intermediate among the tested lithologies. The connected pores lead to a significant decrease in amplitude and a slight increase in time delay under moist conditions.

Purple sandstone is composed of a quartz/plagioclase framework interbedded with mica, chlorite, and other clay minerals. CT imaging shows a relatively dense macrostructure, but SEM observations reveal abundant secondary clay growth that substantially modifies the original intergranular pores, forming numerous micron-scale voids and narrow, tortuous pore throats. The inherent hygroscopicity and resultant interfacial polarization of clay minerals exacerbate energy dissipation and scattering, manifesting as increased attenuation and waveform broadening.

Granite contains a high proportion of feldspar, along with quartz and minor mica. SEM images show interlocking polycrystalline grains with uneven and stepped grain boundaries, while CT slices display strong heterogeneity. The high density of phase interfaces results in intense interfacial scattering and internal reflection, leading to the lowest initial amplitude, fastest attenuation, and greatest waveform broadening. The specimens are highly sensitive to thickness, and the time delay increases markedly with greater thickness.

Overall, mineral composition and internal structure jointly determine the THz transmission capacity and moisture sensitivity of rocks. From a mineralogical standpoint, carbonate-rich, densely crystalline, and compositionally uniform rocks generally exhibit higher refractive indices, leading to greater time delay per unit thickness. In contrast, silicate rocks with diverse mineral phases show moderate time-delay behavior. Hydrophilic minerals such as clay and mica enhance energy loss through water adsorption and interfacial polarization, amplifying attenuation and broadening under moist conditions even when macropores are limited.

Regarding the structural effect, the interface density, grain size relative to the THz wavelength, pore-throat geometry and roughness, and the development of microfractures collectively control scattering and dispersion intensity. Higher interface density and roughness accelerate amplitude attenuation and waveform broadening, whereas connected pore networks facilitate water migration, typically resulting in reduced amplitude and increased time delay.

## 4. Conclusions

Through a systematic series of THz-TDS experiments combined with microstructural analysis, this study clarified the influencing patterns and key factors governing the THz transmission behavior of natural construction rock specimens under different moisture states. The main conclusions are as follows:

(1) Lithology and thickness exert a significant influence on THz wave propagation characteristics. As the specimen thickness increases from 2 mm to 5 mm, all four lithologies exhibit a consistent trend of peak amplitude attenuation, increased time delay, and waveform broadening. These behaviors indicate that greater thickness extends the propagation path and intensifies multiple scattering. Limestone shows the highest amplitude and the longest delay; granite, with the highest density, exhibits the strongest energy attenuation and most pronounced waveform distortion; sandstone and purple sandstone fall between the two, showing lower density, looser structures, and faster propagation velocities.
(2) Moisture exerts the strongest suppression on THz transmission in thinner specimens, while the attenuation becomes gentler with increasing thickness. The relationship between peak amplitude and thickness is well described by the Beer–Lambert exponential decay model. The attenuation coefficient quantitatively characterizes energy loss during the drying–wetting process: granite exhibits the highest attenuation coefficient, limestone the lowest and most stable, while sandstone and purple sandstone lie between.
(3) The THz planar transmission imaging reveals distinct lithologic and moisture-dependent patterns. Limestone exhibits a dense and uniform structure, with stable amplitude distribution and minor irregularity caused by limited pores. Sandstone and purple sandstone display parallel trends in the statistical curves of amplitude parameters, suggesting relatively uniform pore distribution. Granite, due to its poor transmissivity, shows the most pronounced imaging contrast under different moisture states, though thickness remains the dominant controlling factor.
(4) The THz transmission characteristics of rocks are jointly governed by mineral composition and internal structure. Dense, single-phase carbonate rocks (e.g., limestone) show high transmission, low attenuation, and strong refraction; granite experiences enhanced energy loss and waveform broadening due to scattering at grain boundaries and fractures; clay-rich lithologies such as purple sandstone are most sensitive to moisture variation owing to water adsorption and interfacial polarization effects.

As a pioneering exploration of THz technology for the NDT of rock materials, this study establishes a quantitative framework for the structural health monitoring of geomaterials. Future research will focus on developing interpretable mechanistic models based on effective medium theory to further advance the precision of in situ moisture–structure characterization.

## Figures and Tables

**Table 1:** Mass and bulk density of specimens across treatments and thicknesses.

Lithology	Sample Index	Real Thickness/mm	Mass (g)					Density (g/cm^3^)				
			Natural	Initial Drying	Initial Soaking	Secondary Soaking	Secondary Drying	Natural	Initial Drying	Initial Soaking	Secondary Soaking	Secondary Drying
Purple sandstone	PS-T2	2.138	9.364	9.326	9.812	9.870	9.270	2384.523	2374.846	2498.605	2513.375	2360.586
	PS-T3	3.190	14.135	14.078	14.757	14.868	14.003	2399.632	2389.956	2505.226	2524.070	2377.223
	PS-T4	4.166	18.915	18.857	19.838	19.954	18.762	2408.333	2400.948	2525.853	2540.622	2388.852
	PS-T5	5.187	23.646	23.588	24.838	24.976	23.453	2408.562	2402.654	2529.978	2544.034	2388.903
Granite	GR-T2	2.287	11.110	11.101	11.142	11.161	11.098	2829.138	2826.846	2837.287	2842.125	2826.082
	GR-T3	3.127	16.696	16.692	16.740	16.754	16.680	2834.401	2833.722	2841.871	2844.247	2831.685
	GR-T4	4.115	22.309	22.306	22.359	22.366	22.284	2840.470	2840.088	2846.836	2847.728	2837.287
	GR-T5	5.206	27.862	27.859	27.931	27.939	27.837	2838.000	2837.694	2845.028	2845.843	2835.454
Limestone	LS-T2	2.171	10.392	10.391	10.412	10.421	10.390	2646.301	2646.046	2651.394	2653.686	2645.792
	LS-T3	3.165	15.649	15.648	15.672	15.679	15.638	2656.657	2656.487	2660.561	2661.750	2654.789
	LS-T4	4.182	20.899	20.898	20.914	20.932	20.887	2660.943	2660.816	2662.853	2665.145	2659.415
	LS-T5	5.185	26.181	26.180	26.201	26.210	26.169	2666.775	2666.673	2668.812	2669.729	2665.552
Sandstone	SS-T2	2.197	10.605	10.569	10.642	10.678	10.496	2700.541	2691.374	2709.963	2719.130	2672.784
	SS-T3	3.133	15.981	15.905	16.058	16.096	15.790	2713.019	2700.117	2726.091	2732.542	2680.594
	SS-T4	4.184	21.338	21.262	21.472	21.510	21.109	2716.839	2707.162	2733.900	2738.738	2687.681
	SS-T5	5.176	26.681	26.623	26.874	26.913	26.430	2717.704	2711.797	2737.363	2741.336	2692.138

## Data Availability

The original contributions presented in this study are included in the article. Further inquiries can be directed to the corresponding author.

## Abbreviations

The following abbreviations are used in this manuscript:

THz	Terahertz
TDS	Time-Domain Spectroscopy
THz-TDS	Terahertz Time-Domain Spectroscopy
NDT	Non-Destructive Testing
CT	Computed Tomography
NMR	Nuclear Magnetic Resonance
SEM	Scanning Electron Microscopy
XRD	X-ray Diffraction
ERT	Electrical Resistivity Tomography
PCA	Principal Component Analysis
SVM	Support Vector Machine
